# Isolated Endobronchial Metastasis of Breast Cancer Successfully Managed With Multimodal Treatment

**DOI:** 10.7759/cureus.49891

**Published:** 2023-12-04

**Authors:** Ghizlane Rais, Meryem Maskrout, Rania Mokfi, Fadoua Rais, Hind Serhane

**Affiliations:** 1 Medical Oncology Department, CHU (Centre Hospitalo-Universitaire) Souss Massa, Agadir, MAR; 2 Oncology Department, Biomed Laboratory, Faculty of Medicine and Pharmacy of Agadir, Ibn Zohr University, Agadir, MAR; 3 Oncology Department, Ibn Zohr University, Medical School of Agadir, Agadir, MAR; 4 Radiation Therapy Department, University Hospital Center of Montreal, Montreal, CAN; 5 Pulmonology Department, CHU (Centre Hospitalo-Universitaire) Souss Massa, Agadir, MAR

**Keywords:** case report, multimodal treatment, cdk4/6 inhibitor, breast cancer, endobronchial metastasis

## Abstract

Isolated endobronchial metastases of breast cancers, without other visceral metastatic involvement, are exceptional. We report here an observation of isolated endobronchial metastasis discovered 18 months after complete treatment of breast carcinoma. The endobronchial metastasis was revealed by an incoercible cough and hemoptysis. A bronchoscopy revealed a budding tumor process obstructing the right stem bronchus and a biopsy was performed. The anatomopathological and immunohistochemical analysis confirmed the metastatic nature of the endobronchial tumor. The patient received treatment with palbociclib and aromatase inhibitors. Two years after radiotherapy and under hormone treatment, the patient is in complete remission of her breast cancer and endobronchial metastasis. Emerging research suggests that oligometastatic breast cancer carries a superior prognosis. We believe that patients with oligometastatic breast cancer should be treated with curative intent, including ablative therapy to all sites of disease if it can be safely accomplished. This approach may offer an additional chance for prolonged survival.

## Introduction

While secondary pulmonary parenchymal metastasis is quite frequent, endobronchial metastases of breast cancers are extremely rare [1]. Only 0.4% to 1% of patients with breast cancer present this type of lesion whether at diagnosis or after relapse, which raises the question of its primary or secondary origin [2,3].

Common sites of breast cancer metastases are liver, lung, bone, and central nervous system. Less common sites include the gastrointestinal (GI) tract, pancreas, thyroid, spleen, pituitary, abdominal lymph nodes, skin, and oral cavity. Although distant metastases are informative of the aggressive nature of the primary, oligometastatic breast cancer (meaning less than five metastases) represents an intermediate prognosis between locally advanced and widely metastatic disease [4]. In view of its more limited capacity for widespread extension, oligometastatic disease is thought to benefit more from aggressive ablative treatment of known metastases. A recent research by Nesbit et al. suggests that oligometastatic cancers have a more favorable prognosis. Hereby, we provide a brief review of the existing literature regarding endobronchial metastasis of breast cancer as well as the proposed treatments for oligometastatic diseases [5].

## Case presentation

We report here a case of a 54-year-old postmenopausal woman with no significant past medical history, who presented with a one-year history of a neglected right breast mass. Physical examination revealed an irregular mass on her right breast associated with a round erythematous plaque and several palpable axillary lymph nodes. Subsequently, a mammogram revealed a spiculated mass with diffuse skin thickening throughout the whole right breast. No abnormalities were detected in the left breast. An ultrasound revealed an ill-defined hypoechoic mass of 46 mm in the upper outer quadrant of the right breast. Two abnormal axillary nodes were also found. Afterward, a micro biopsy of the breast mass was performed, revealing a grade III invasive carcinoma of nonspecific type (NST), with positive estrogen (90%) and progesterone (90%) receptors (ER and PR, respectively), and negative HER2 (estimated score of 1). Further assessment, made of thoracoabdominal and pelvic CT scans as well as bone scans revealed no evidence of systemic metastases.

According to the multidisciplinary team decision, the patient received systemic neoadjuvant treatment for four AC60 cycles followed by weekly paclitaxel for 12 weeks. An incomplete clinical response was observed. Subsequently, a right mastectomy with axillary dissection was performed. Pathological examination showed no pathologically complete response with a 3 cm tumor residue made of grade II, invasive carcinoma without any specific type and four positive lymph nodes. Thereafter, adjuvant radiotherapy to the right chest along with the supraclavicular nodal area was done. She was then started on antiestrogen therapy with letrozole.

**Figure 1:**
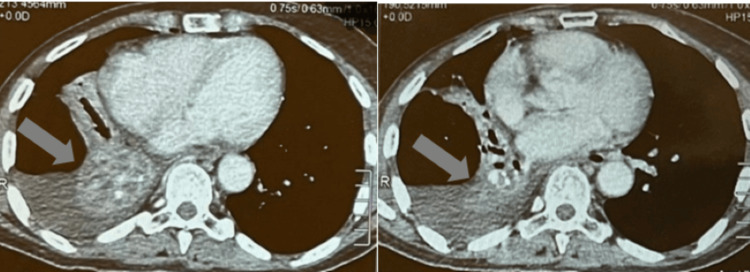
Chest CT scan showing a right lower lobe atelectasis.

Eighteen months later, the patient showed up with a persistent cough and hemoptysis. A chest CT scan was performed and revealed a right lower lobe mass (Figure 1).

**Figure 2:**
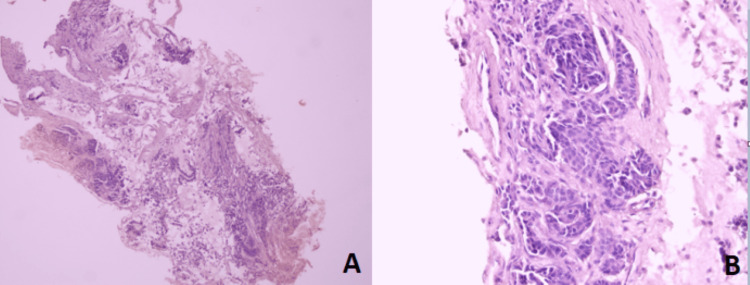
Anatomopathological examination of the endobronchial mass showing poorly differentiated tumor proliferation with hematoxylin and eosin (H&E) staining at low (A) and high magnification (B).

**Figure 3:**
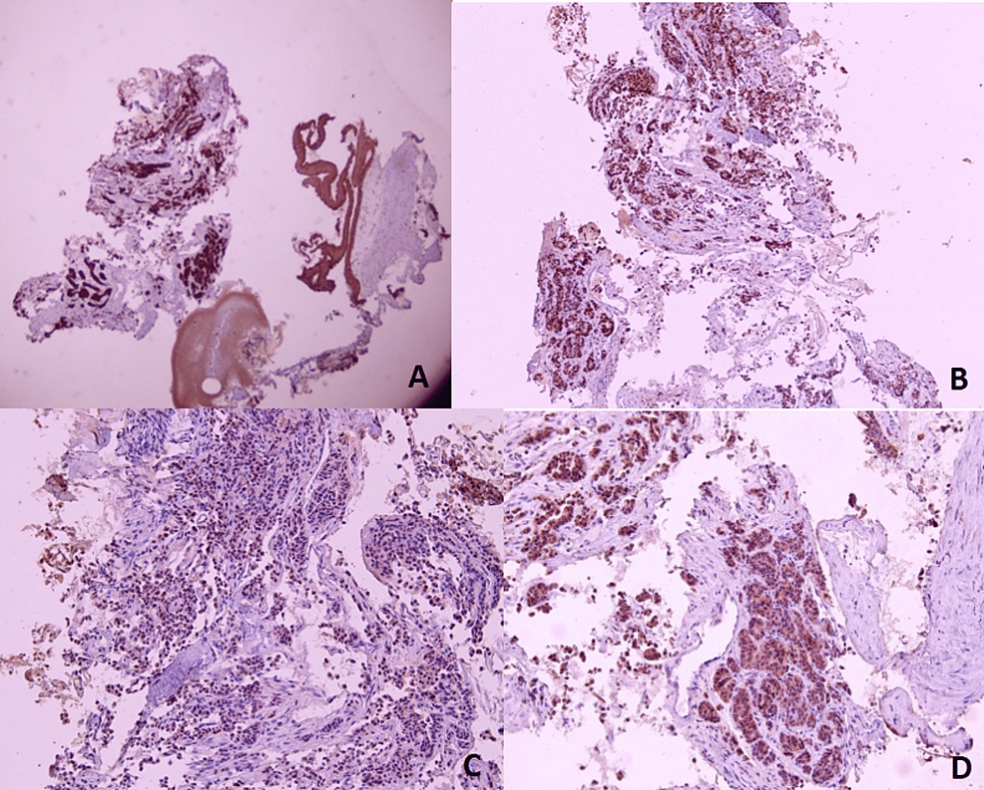
Immunohistochemical study showing that tumor cells express cytokeratin AE1/AE3 (A), estrogen receptor (B), and progesterone receptor (C) but do not express thyroid transcription factor 1 (TTF-1) (D).

Bronchoscopic examination showed a pedunculated mass in the right middle lobe bronchus. Then, biopsies with pathological examination revealed a poorly differentiated tumoral proliferation (Figure 2) with an immunohistochemical profile compatible with an endobronchial metastatic site of invasive breast carcinoma that was 90% positive for estrogen receptor (ER), 60% positive for progesterone receptor (PR) and negative for thyroid transcription factor 1 (TTF-1) (Figure 3).

**Figure 4:**
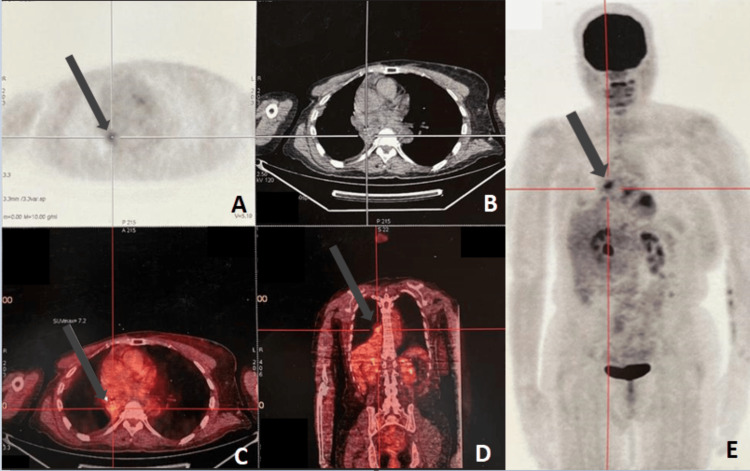
PET/CT scan showing high fluorodeoxyglucose uptake in the right lower lobe bronchus (maximum standard uptake value = 7.2) (A-D) with no other hypermetabolic sites (E).

To look for other potential metastatic sites, a whole body 18F-fluoro-2-deoxyglucose PET-CT scan was performed and showed high fluorodeoxyglucose (FDG) uptake in the right lower lobe bronchus (maximum standard uptake value of 7.2), with no other hypermetabolic uptakes (Figure 4). Therefore, the patient was deemed to be in an oligometastatic relapse.

**Figure 5:**
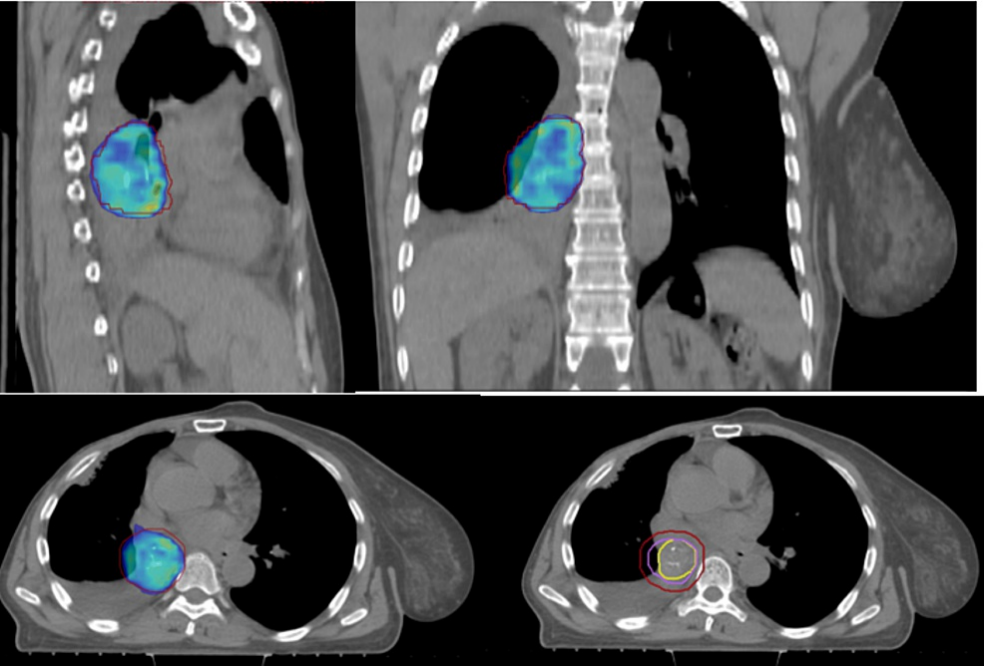
Images showing radiotherapy plans.

After the case discussion in the multidisciplinary tumor committee meeting, and considering the proximal endobronchial metastasis location, a pneumonectomy was proposed, but unfortunately, the patient refused any surgical intervention. It was therefore decided to perform intensity-modulated radiation therapy (IMRT) since stereotactic radiotherapy could not be considered in our context of an underequipped regional center (Figure 5).

Subsequently, she received the CDK4/6 palbociclib inhibitor combined with exemestane. Endoscopic control revealed a complete response. With a follow-up period of 27 months after the end of her radiotherapy, the patient sustained good tumor control under the ongoing hormone treatment.

## Discussion

Common malignancies that metastasize to the trachea and/or bronchial tree include lung cancer as well as renal and colorectal cancers and lymphomas. The frequency of endobronchial metastasis (EBM) from non-pulmonary malignancies is quite variable ranging from about 2% to 50% [4].

Breast cancer is likely to metastasize relatively early to the regional lymph nodes and thereafter primarily to the lungs, red bone marrow, liver, and bone [5]. EBMs of breast cancer are uncommon occurring in about 1% of patients [3]. However, isolated endobronchial metastases of breast cancers, without other visceral metastatic involvement, are exceptional.

The average age of diagnosis is 55 years (39-71). It is usually delayed by the lack of symptoms or evidence of metastatic disease elsewhere [6]. In our case, the patient was 54 years old.

Lung metastases from breast cancer usually occur within two to three years of the disease course. However, cases of early (six months) or delayed onset (33 years) have been reported [7]. EBM may appear during the follow-up of a patient known to be a carrier of the disease or may reveal cancer [2]. In our case, the delay was 18 months after the end of adjuvant radiotherapy and the start of hormone therapy.

Breast cancer thoracic metastases appear as solitary or multiple metastases, including pulmonary nodes, endobronchial metastases, lymphangitis carcinomatosis, pleural metastases, lymph node involvement, as well as skeletal metastases [8]. Modes of metastasis involve bronchial invasion by a parenchymal lesion, direct metastasis to the bronchus, bronchial invasion by mediastinal or hilar lymph node metastases, and endobronchial invasion with lymphangitis carcinomatosis [3].

Diagnosis of endobronchial involvement with breast cancer requires a high index of suspicion. Several studies reported that asymptomatic patients range from 20% to 62.5% [9]. Among clinical symptoms, cough and hemoptysis are the most common presenting symptoms, which occurred in 71% and 25% of patients, respectively [8].

Chest X-ray findings are mostly abnormal, frequent, and nonspecific. They show the endobronchial mass, which is often associated with obstructive atelectasis (58%) with combined pneumonitis [10].

Fluorodeoxyglucose PET (FDG-PET) can be useful for the diagnosis of secondary malignancy of the tracheobronchial tree and to distinguish it from vascular structures as well as other benign lesions [11]. Thus, nodes smaller than 6 mm cannot be adequately evaluated with this modality [12]. It is also of added value to rule out the existence of other metastatic sites.

Flexible tracheobronchoscopy with biopsy is a valuable tool for the definitive diagnosis of EBM. The endobronchial appearance consists generally of mucosal thickening and edema. The tumor involves the submucosal lymphatics rather than the mucosa surface. This could explain why bronchial cytology is rarely positive and emphasizes the need for a relatively deep biopsy [13]. Moreover, in some clinical settings, bronchoscopic and pathologic findings may not allow to distinguish the primary from metastatic tumors [14]. Endobronchial histologic specimen review along with those from the extrathoracic primary tumor is of paramount importance to reach the correct diagnosis and should be performed in all cases with a history of previous malignant disease [10].

Immunohistochemical staining is crucial to determine the primary tumor site. It should be performed systematically in all such cases to look for similar morphologic and immunoreactivity features between the primary and recipient tumors to retain the diagnosis. Furthermore, the expression of TTF-1 is among the most specific markers to distinguish primary pulmonary adenocarcinomas from other metastases [9].

In our case, it is important to underline that the diagnosis of EBM of breast cancer was retained on a bronchoscopy with biopsy. Histological and immunohistochemical characteristics of luminal breast carcinoma were documented, thus we concluded that the breast carcinoma was the cause of the right bronchial metastasis.

When the EBM is unique after a comprehensive metastasis workup, including FDG-PET/CT scan and brain MRI, the disease can be considered oligometastatic [5]. Given its restricted potential for extensive spread, aggressive ablative therapy targeting identified metastases may be advantageous for oligometastatic disease. There are several options for ablation, including radiofrequency, surgery, hypo-fractionated radiotherapy, and brachytherapy.

In the specific cases where EBM represents the only site of metastases, surgery could be considered - if technically feasible - since good results and long progression-free survival have been reported in certain series using this approach [15]. The criteria to select patients who are good candidates for surgical resection of EBM are like those with lung metastases, including technical resectability, tolerable general and functional surgical risk, good control of the primary process, and the exclusion of any further extrathoracic metastases. The standard and necessary procedure is anatomic resection such as segmentectomy or lobectomy. Extended resection with or without lymph node dissection was not deemed necessary for most studies [16].

Radiotherapy could be used when surgery is not an option. Unfortunately, the dose required for effective tumor control exceeds the normal lung tissue tolerance [17]. Indeed, stereotactic radiotherapy has been proposed as a good alternative, to overcome the traditional radiotherapy limitations, especially when tumors are functionally unresectable [17]. Stereotactic body radiotherapy (SBRT) and IMRT seem to be safe and associated with good outcomes. In several studies, patients with oligometastatic breast cancer and well-controlled primary were treated with SBRT targeting all metastatic sites, and progression-free survival ranged from 50% to 70% at two years [18,19]. The optimal ablative therapy for endobronchial oligometastases should ideally be decided in a multidisciplinary meeting including surgeons, radiation oncologists, and interventional radiologists’ input [18,19].

For more extended tumors, systemic treatment is the most effective treatment. The choice of treatment regimens depends on the tumor type, molecular biomarkers, disease extent, and the previously administered treatments [16]. For luminal breast cancer, a key point in the treatment is maintaining hormone and target therapy, and reserving chemotherapy to the setting of visceral crisis [17]. CDK4/6 inhibitors have become the standard of care in first-line treatment of metastatic luminal breast cancer [20]. With such an approach, even if the metastatic disease cannot be completely cured, it is possible to stabilize it for a long period.

Considering our patient’s hormone sensitivity and her metastatic relapse status, treatment with CDK4/6 plus aromatase inhibitor was indicated. This also implies that, for our curatively treated patient as well as for other oligometastatic patients, both disease and therapy burden must be considered to choose the optimal medical treatment regimen, ideally by a team with extensive clinical expertise in this area.

Bulky lesions of the main and lobar bronchi may benefit from endobronchial treatment. Endobronchial resection can be performed using rigid bronchoscopy to provide adequate ventilation for patients with palliative intent. Nd-YAG laser can be useful for hemostatic control when needed. Once the endoscopic debulking is completed, additional cryotherapy or stent insertion can be considered. In case of carina or trachea involvement with asphyxia risk, interventional bronchoscopy may be considered with further possibility of stent placement [16].

There is general agreement that EBMs represent an advanced stage of disease with poor prognosis. However, oligometastatic breast cancer can receive curative treatment with superior outcomes. In a recent meta-analysis, including 1937 patients with isolated lung metastases, a five-year overall survival as high as 46% was reported. In these cases, the main favorable prognostic factors were a status of solitary metastasis, complete metastases resection, longer disease-free interval (DFI), and positive hormone-receptor disease [19].

## Conclusions

In conclusion, solitary endobronchial metastatic occurrence during breast cancer progression is a rare situation. Considering the long latency period of EBM, patients with prolonged and refractory pulmonary symptoms associated with higher EBM risk should be candidates for bronchoscopy. Treatment options for EBM, especially in the case of a unique site of metastasis, should be patient-tailored in a multidisciplinary setting.

The current literature review suggests that most patients with oligometastatic breast cancer should receive curative treatment, including ablative therapy of all disease sites, as long as this can be achieved safely.
